# History of Islamic Medical Schools in Turkey’s Territory

**DOI:** 10.4274/balkanmedj.galenos.2020.2020.4.160

**Published:** 2020-10-23

**Authors:** Selman Çıkmaz, Recep Mesut

**Affiliations:** 1 Department of Anatomy, Trakya University School of Medicine, Edirne, Turkey; 2 Emeritus Prof. Dr.

**Keywords:** Historical hospital buildings, islamic physician training institutions, islamic foundations, medical history

## Abstract

In today’s political borders of the Republic of Turkey, there exist very old institutions that train physicians according to Islamic medical science. In this study, 19 health institutions whose locations have been determined and documents finalized were approached in a chronological order and classified according to the historical periods: XII^th^ and XIII^th^ centuries (Seljukian period)-10, XIV^th^ century (Ilkhanate dominion)-1, and XV^th^-XVII^th^ centuries (Ottoman period)-8 institutions. Some of them have a history of 900 years (Konya Mâristan-ı Atik, 1113; and Mardin Eminüddin Bimaristanı, 1122). In addition, some are in the form of a medical madrasah and an application hospital (Kayseri, 1206; Sivas, 1217). In these institutions, great masters of Islamic medicine (Razi, Fârâbî, Bîrûnî, İbni Sina) and ancient authorities (Hippocrates, Dioscorides, Aretaeus, Galenos) were taught. These institutions had builders, rulers (sultan, melik) or mothers, wives, daughters, and sisters (the presence of female builders in these institutions attracted attention). During the Seljuk period, powerful viziers also built such institutions. These hospitals also provided free services which were considered as “charities” according to the Islamic religion. These institutions were financed by sources (shops, inns, Turkish baths, bridges, mills, vineyards, gardens, fields and annual taxes of many villages) that donated funds through the “foundation” method. Donations were made in the presence of the “kadi” (muslim judges) and many witnesses, with the written document “endowment.” These foundations were not touched by subsequent monarchs. Payment of fees, daily expenses of the physicians, assistant personel and repairing of buildings was done by the board of trustees. Twelve of these institutions are still in use for public interest (polyclinic, museum, health museum, library, university, and education center). When modern medical schools (1827) and hospitals (1842) began to be established as of the XIX^th^ century, these historic buildings were allocated to mental patients, while some were devastated by neglect. However, in the Republic period, they have been restored and used for health and educational purposes.

This study covers the oldest Islamic institutions that trained physicians according to islamic medical science in today’s political borders of the Republic of Turkey. These medical schools can be classified to three historical periods: I. Early period: Seljuks period-XII^th^ and XIII^th^ centuries. These vassal statesmen who were established under the Great Seljuk Empire (1038-1194) were then subjected to Konya (Iconon) Seljuk Sultanate (1074-1308) in the center of Anatolia and were gradually annexed and eliminated by this Sultanate.

II. The intermediate period: The period of the reign of İlhanlı (İlkhanid)-XIVthcentury. As a result of the Great Mongolian invasion, the İlhanlı State (İlkhanid, 1256-1335), founded by Hulagu Khan the grandson of Genghis Khan based in Tabriz, had placed Konya Seljuks and other principalities in Anatolia under its guardianship and administrated them by means of a general governor in Amasya (Amasseia).

III. Late period: Ottoman period-XV^th^-XVII^th^ centuries. The Ottoman Principality, sprouting in the northwestern corner of Anatolia, benefiting from the weaknesses of the Byzantine in the west and Seljuk and İlkhanid in the east, conquered the Balkan Peninsula and İstanbul (Constantinopolis), and eventually became a powerful empire by annexing all the states in Anatolia (1299-1923).

Islam was accepted by the Turkish speaking communities in Central Asia from the X^th^ century and spread through the Iranian territory to the Middle East and Asia Minor (Anatolia) in the XI^th^ century. The Great Seljuk Sultan Alparslan destroyed the Byzantine army in the Battle of Manzikert in 1071 and captured the Emperor Romanos Diogenesn IV. When all Asia Minor was defenseless and helpless, the commanders sent by Alparslan gradually turned to the west and dominated the lands of Eastern Anatolia and Central Anatolia (1). Since the beginning of the XIIth century, the independent Konya Seljuk Sultanate and some vassal emirates (kings) have built enormous and durable buildings by establishing educational, health, and social institutions according to Islamic traditions, some of which have survived till present day (2). Architectural designs in Baghdad, Damascus, and Aleppo were taken as examples; knowledgeable and experienced physicians were invited; Abu Bakr er-Râzî (Rhazes, 854-925), Fârâbî (Alpharabius, 872-950), Bîrûnî (Alberuni, 973-1050) and Ibni Sina (Avicenna, 980-1037) books were read as the main opus (3).

Considering that civilization has been very old in these lands (Göbeklitepe, 10,000 years BC), the medical knowledge is very old as well. The most famous physicians and pharmacists of ancient times grew up in Anatolia, absorbed and systematized the accumulation of the generations before them, and became the founders of medicine, dentistry, and pharmacy education in the world. These rudiments were not destroyed when the Muslim Arabs conquered Eastern Anatolia in the VII^th^ century AD. On the contrary, Greek, Latin, Syriac, Armenian, and Hebrew medical books have been translated into Arabic and Persian for the benefit of the physicians. An extraordinary exchange of information and synthesis has emerged in this multilingual and multi-religious society that lived together in harmony. Bukrat (Hippocrates of Cos, 460-377 BC), who is of Western Anatolian origin and accepted as the “Father of Medicine” and Calinus (Galenos of Pergamon, 130-210), who greatly influenced Islamic Medicine, were accepted as undisputed authorities and thoughts (3,4). The great clinician Arete (Aretaeus of Cappadocia, 80-138), who was born in Kayseri (Caesarea) and Skoridos (Pedanius Dioscorides of Anazarbus, 40-90), who was born and raised on the lands of Çukurova (Cilicia) have taken their respected places in (5).

Rational arrangements were made in the field of public health and the most advanced medical knowledge transferred to young physicians. A “head physician” (chief doctor, melik-ül-etıbba, şeyh-ül-hükema), who was the equivalent of the Minister of Health, made the appointments, transfers, and investigations of the physicians and subjected them to the “false physicians” examination. Builders of these physician-educating organizations became either rulers (“sultan” or “melik”) or made buildings on behalf of their mothers, wives, or daughters. It is worth noting that female builders existed within an islamic society. As the sense of compassion of women was more evident in the care of patients, the power and reputation of women was at the highest level in these first Turkish states.

A “foundation” was established according to the Islamic principles in order to guarantee the needs and expenses of these institutions for many years and an official written document (“endowment”) was issued. Taxes of real estates (inns, baths, bridges, shops, fields) that yielded income through local endowments that were regulated in the presence of the local “kadi” and witnesses, as well as the taxes from many villages were donated and the board of trustees of the foundation were granted powers. The future of these “charities” made for religion and humanity were secured so well that these facilities operated until the XIXth century and their physical spaces were still partially standing. These health facilities that were established as charities provided free services; patients who received an outpatient exam or inpatient treatment were given free medication. No discrimination based on race, language, religion, sect, or gender had been made in this regard. Three meals, hospitalization, heating, and lighting expenses of the inpatients were also covered by the foundation. The foundation also paid the salaries of the chief physician, other physicians, “kehhal” (ophthalmologist), surgeons, and pharmacists (staff-accounting-teller, cleaning and maintenance staff). Daily medical allowance was given to medical students (“Şakird”) who received free boarding education.

Subsequent Islamic rulers (Ottomans) did not touch the foundation’s properties and businesses that were donated before their time, approved the endowments, and also contributed materially and morally. The Ottoman Sultans (their mothers, wives, and daughters as well) also continued the same tradition in the Western Anatolia and European lands (Rumelia), who were later Islamized, and established and dedicated new health and welfare institutions. Although a portion of these endowments were lost, most of them have survived until today and are safeguarded by the General Directorate for Foundations of the Republic of Turkey. Information can be gathered from the rich archive of the Ottoman State, which has kept extraordinary bureaucratic, meticulous, and detailed records about those that disappeared.

In terms of the traing of physicians, we can divided these institutions into two:

1) “Master-apprentice” model: Young candidates (“apprentice”) starting from scratch sat under an experienced physician (“master”) who had made a name for himself in inpatient treatment institutions that carried out diagnosis and treatment of patients, and learned the secrets of the profession by observing and questioning for years. They personally undertook studies in the areas of patient care, preparation and administration of medications, and discussed theoretical knowledge by reading the books given to them by their master. This included one or more people, their training period was individually determined by the master (not less than three years), and they would eventually receive “consent” (qualification) according to the personal opinion of their master. Different names have been used in Anatolia for this type of inpatient treatment institutions: “bimaristan” (Persian “bimar” = patient + “-istan” = place) or just “maristan” “Şifahane” (Arabic “şifa” = healing + Persian “-hane” = “home”) and compound terms derived from “dâr-üş-şifa” (home of healing), “dâr-üs-Sıha” (home of health), “Dar-ül-afiye” (home of well-being). In the Ottoman period, the term “dar-üt-tıbb” (medical dormitory) was also used. The term “Hastahane” (hospital) is quite new (1845, Bezmiâlem Gureba Hastanesi).

2) "Medrese” (madrasah) model: It was different from general madrasahs (theology, philosophy, law, administration) and specialized higher education institutions were formed to train only physicians. They taught more students (10 and more), “lessons” (→ madrasah) were taught by more than one teacher; general culture, oratory and foreign language (Arabic and Persian) were taught as well. These were called “medrese-i etıbba” (faculty of medicine). Adjacent to these, there was always an application hospital (darüşşifa). In some cases, a general madrasah and a medical madrasah were built side-by-side (double madrasahs). The education period would be between four and six years, exams were applied, and the sultanate possessors would attend the graduation ceremonies. Graduates were given diplomas.

## EARLY PERIOD (SELJUK PERIOD)

The biggest Turkish state established in Anatolia at the end of the XI^th^ century was the Konya Seljuk Sultanate. They became relatives with the Great Seljuk Dynasty in Iran (uncle’s sons) and were able to unite smaller emirates with their military superiority and spiritual reputation, as well as their strategic positions and rational governments in the center of Anatolia. They also took the Black Sea (Sinop, Synope) and Mediterranean (Antalya, Attaleia) beaches. Seljuk Sultans, who undertook extensive zoning activities in the lands they dominated, built hospitals and training institutions for physicians in the cities of Sivas, Çankırı, Kastamonu, and Tokat, especially the capital city of Konya (6,7).

**Figure 1. A-C:**
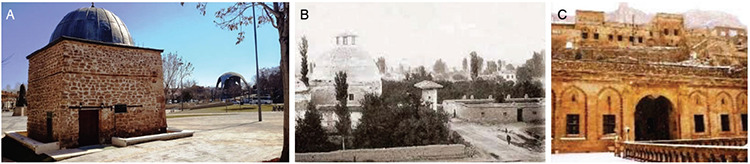
Early period Hospitals: Konya, the masjid of Sultan Alâeddin Darüşşifası, which was rebuilt in 1221 instead of Maristan-ı Atik which was built in 1113 at Sultan Melikşah’s period (after the 2018 restoration) (a) (14); Konya, Küçük Karatay Madrasah (Kemaliye Darüşşfası [hospital]) built in 1254 opposite Büyük Karatay Madrasah (used as a museum today) established in 1251 (photo dated 1960) (b) (15); Mardin, Bimaristan section of Eminüddin Külliyesi (Islamic complex) dated 1122 which remains today in ruins (c) (16).

**A) Konya Hospitals:** Three health institutions started operating in Konya in the XII^th^ and XIII^th ^centuries according to written documents and some archaeological findings:

1) 1113, Mâristan-ı Atik was founded in the period of Sultan Melikşah. According to our present knowledge, it was the oldest medical training institution established in the territory of Turkey: 1 Şaban 506 Hicri (= Gregorian calendar January 21th, 1113). Endowment of this facility was found in the Ottoman Archives and its first translation by Hakkı Öcal was published in 1937 (8). It is believed that today, it is located in the Şifahane Neighborhood in Konya. According to the definition of physicians and officers in the endowment, there are not only treatment services, but also physician training duties (“Şakird-i tabib” is mentioned).

2) 1221 in the period of Sultan Alâeddin Keykubat, the establishment of a second health institution, Alâeddin Dârüşşifası (Dârüşşifa-i Alâî) was implemented and served for many years (until 1858). As a result of invasions and earthquakes during the Crusades, the previous Mâristan-ı Atik, which was established 90 years ago, became unusable and was built once again at the same location by Konya Kadi Izzeddin Muhammed bin Mahmud upon the order of the Sultan. For this reason, it is referred to as “Kadi Izzeddin Mâristanı” in his foundation. However, it was mentioned in the later Ottoman documents as “Konya Alâeddin Dârüşşifası” (9,10). This health facility with its buildings such as complexes, madrasahs, and masjids in a complex style has remained till today with only its masjid in ruins [“Şifahane (Sakahane) Mescidi”] and was repaired and opened to visitors by the Municipality of Konya in 2018 (Figure 1a).

3) 1254 As a third health institution, Kemaliye Dârüşşifası (Karatay-ı Sagîr Madrasah) came into service and its buildings survived until 1960 (Figure 1b). After the famous Seljuk vizier Celâleddin Karatay built the Great Karatay Madrasah (nowadays a Museum) in 1251, he built the Karatay-ı Sagîr (i.e., Küçük Karatay) Madrasah right across it, for medical education and care of patients on a large field in 1254. He appointed his brother, Kemâleddin, who was a physician in his foundation of the same date, as the trustee of this facility. For this reason, this facility which we can call “the first private foundation medical school,” was later called Kemaliye Dârüşşifası (11). Our famous medical historian, who visited Konya in 1956 and 1960: Distinguished Professor Doctor A. Süheyl Ünver saw and narrated about the buildings that were still standing but not used. In his visit in 1972, he regretfully found out that the local administrators were expanding the road passing through the building, which ended up destroying 700 years of historical artifacts.

**B) 1122, Mardin, Eminüddin Hospital:** The southeastern corner of today's Republic of Turkey passed into the hands of Arab Muslims in as early as 640. With the weakening of the Baghdad Caliphate, these lands were left defenseless and after the Manzikert War, the House of Turkmen “Artuklular” (Artuqids) was established under the command of Artuk Bey (1102). Necmeddin İlgazi (1104-1122) was the head of the Mardin segment of this House, which was torn as a result of internal conflicts. His brother Melik-ul-Cebbar Eminüddin (referred to in the Ottoman records as Şeyh Eminüddin) started the construction of a large-scale complex (mosque, madrasah, bimaristan, Turkish bathhouse, fountain) as a charity, but when he died, Necmeddin, who was the ruler, completed it in 1122 (12,13).

It was placed as stepped landings on the sloping land with natural hot healing water springs at the southwest end of the historic city of Mardin, located at the foot of a rocky hill overlooking the northern Mesopotamian plains. It has survived until today in the territory of Turkey as the oldest mosque complex, which benefits from natural water sources that can be counted as a hydrotherapy (spa) center (Figure 1c). Although his endowment did not survive till today, it was mentioned as Eminüddin Bimaristan Foundation in the Ottoman Archives from 1518 until the beginning of the XX^th^ century and his staff had two physicians. Today, the Maristan Turkish Bath and Maristan Fountain are still in use, but the other parts are in ruins.

In the XIII^th^ century, the Turkish states in Anatolia experienced their brightest periods and built many new health institutions besides the rich zoning activities. The Seljuk Sultans personally led the way and equipped large cities (Kayseri, Sivas) outside the capital with imposing madrasahs and hospitals. Viziers and commanders at their disposal have also built health facilities in northern provinces (Çankırı, Kastamonu and Tokat). The people of Mengüjek, who were vassal leaders, also built an extraordinary Cami-Darüşşifa complex in Divriği.

**Figure 2. A-C:**
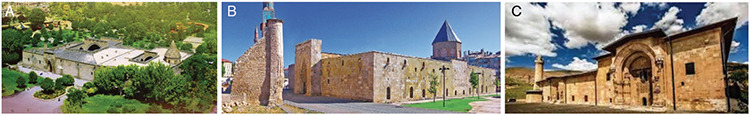
Classical Seljuk Medical Madrasahs: Kayseri, “Çifte Medrese” dated 1206: Gevher Nesibe Darüşşifası (hospital) and Gıyâsiyye Madrasah, “Turkey’s first medical school” (a) (22); Sivas, Sultan İzzeddin Keykâvus I Dârüssıhha (hospital) dated 1217, “Turkey’s second medical school” (b) (23); Divriği (town), Melike Turan Hatun Dârüşşifası (hospital) and Melik Ahmetşah Mosque dated 1228. They are on the UNESCO World Heritage list since 1985 (c) (24).

**C) 1206, Kayseri Gevher Nesibe Hospital:** Sultan Gıyâseddin Keyhüsrev built the “Gevher Nesibe Dârüşşifası ve Tıp Medresesi” in the name of his sister Gevher Nesibe Sultan, who died due to tuberculosis in Kayseri in 1204 (historically Caesarea, the birthplace of Aretaeus) and built “Gıyâsiyye Medresesi” (open courtyard, adjacent “double madasah”) right adjacent to it. The tomb of Gevher Nesibe is also in the madrasah. Eventhough the foundation cannot be found, the Ottoman records (1500,1584) mention professors and students. Until 1890, it carried out its purpose and physicians were trained. According to S. Ünver, “It is Turkey’s first medical school” (17,18). It is the first monumental health facility restored by the government of the Republic and has been acting as a History of Medicine Museum since 1982 (Figure 2A).

**D) 1217, Sivas İzzeddin Keykâvus Hospital:** Izzeddin Keykâvus I, son of Gıyâseddin Keyhüsrev I, came to the throne in 1211 and used Sivas (historically “Sebastea”) as the capital. In 1217, he built a large health institution (“Darüssıhha” or “Şifaiye Medresesi”) on his own behalf. The endowment was found and published in 1938; this was a training school for physicians and was characterized as “Turkey's second medical school” by S. Ünver (19,20). The tomb of the Sultan, who died of tuberculosis in 1220, was also here. This architectural work, with an open courtyard, portico, and three iwans, has also been renovated and opened to visitors (Figure 2B).

**E) 1228, Divriği Grand Mosque and Hospital:** The smallest House established in Anatolia was in the House of Mengüjek (1080-1252). Theb Divriği branch of this House, which was divided into two in 1142, brought a very exceptional work to our history; Mosque and Hospital complex adjacent to each other. In Hijri calendar 626 (Georgian calendar 1228), Ulu Camii (Great Mosque) on behalf of the Divriği judge Melik Ahmetşah and Darüşşifa (Hospital) on behalf of his wife Melike Turan Hatun were built, survived to the present day, and have been on the UNESCO World Heritage list since 1985. There is also the tomb of the monarch family (15 sarcophagi) within the Hospital. Although the original foundation of 1243 is no longer present, the Mahmut Bey foundation of 1397 is attributed to the said foundation and informs about the Hospital staff. The hospital in Divriği, which lost its importance in the Ottoman period, was used as a general madrasah (21). Apart from the main roads, Divriği, which is in a mountainous area that is difficult to reach, is a small town today (10,000 population, altitude 1225 m), but thanks to the Ulu Camii-Darüşşifa complex, which is a masterpiece of stonemasonry, it invites architects and medical historians from all over the world (Figure 2C).

Regional administrators working under the command of the Konya Seljuk Sultans with the title of “atabey” or “pervâne” also did not fall behind and built health facilities as charities (25,26).

**Figure 3. A-C:**
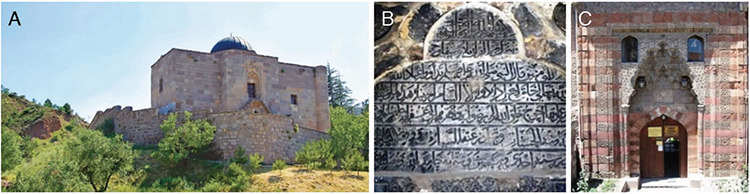
Medical facilities built by Seljuk viziers: Çankırı, 1235. “Taşmescid” (Darülhadis and tomb) which remained from Atabey Cemaleddin Ferruh Darülafiyesi (hospital) (a) (31); Kastamonu, 1272. Epitaph remaining from Pervaneoğlu Ali Bey Darüşşifası (hospital) (b) (32); Tokat, 1277. Crown gate (portal) of Pervane Muineddin Süleyman’s “Çifte Medrese” (c) (33).

**F) 1235, Çankırı Atabek Cemâleddin Hospital:** Atabey Cemâleddin Ferruh, who was the trustee of Sivas Darussıhası, built an interesting complex in his name in Çankırı (formerly Gangra city of Galatia). According to his inscription, in the year H. 633 (1235 AD), he added a “Darülâfiye” for patients and 7 years later, a two storey building adjacent to the north (in 1242): the upper floor Darülhadis, the lower floor Mausoleum. In the 1940s, Süheyl Ünver witnessed the Şifahane part (hospital) in an unrecognizable ruin. It was being used as a Mevlevihane (mevlevi lodge) with wooden additions (27). Darülhadis and its tomb, which are known as “Taşmescid” today, were restored by the Special Administration in 2011, and the foundations of Darülafiye were revealed by excavation (Figure 3A).

**G) 1272, Kastamonu Pervâneoğlu Ali Bey Hospital (Kastamonu Hospital):** Ali Bey, the son of the famous Seljuk vizier Pervâne Muîneddin Süleyman, built a comprehensive complex in the city of Kastamonu (Timonion of Paphlagonia) in 671 (M. 1272): Dârüşşifa, Mosque, Imaret, Library, Mausoleum, and two fountains. The original Hospital building was burned down in 1837; only its inscription on the portal door was left buried on the ground (Figure 3B) and a sidewall has survived to the present day. Known as the “Yılan Külliyesi” (Snake Complex) (due to the snake reliefs as a health symbol), the building was used by the Kadiri sheikhs until the lodge and hermitages were closed (1935). The General Directorate of Foundations restored the Yılanlı Camii, Abdülfettah-ı Veli Türbesi (tomb), and fountains in 2009 (28).

**H) 1277, Tokat Muîneddin Süleyman Hospital:** In the years when the Konya Seljuk Sultanate came under the guardianship of the Mongols, Pervâne Muîneddin Süleyman, an influential politician, also built a magnificent “double madrasah” in the city of Tokat (former Comana Pontica) with two iwans and open courtyards. Although neither its foundation nor its inscription has survived, the main building undergoing repairs stands in the city center under the name “Gök Medrese” (or “Kırkkızlar Medresesi”) and has been used as a Museum since 1926 (Figure 3C). It is a typical example of the Anatolian Seljuk architecture. When the banned Muîneddin Pervane, who was accused of treason, was put to trial and executed by the Mongols on August 2^nd^ 1277, it was completed by the daughter of Konya Seljuk Sultan Gıyaseddin Keyhüsrev III, who was his spouse (29,30).

## INTERMEDIATE PERIOD (ILKHANID SOVEREIGNTY PERIOD)

The first half of the XIV^th^ century was difficult for the Anatolian Turkish states under the exploitation of the Mongols and many powerless houses struggling for existence could not engage in large-scale development activities. However, during the Gazan Khan period (1295-1304), the Ilkhanians accepted Islam and started building charitable institutions belonging to the Islamic civilization. An important Darüşşifa (hospital) was built in Amasya (Amasseia, the capital of the historical Pontus state and the birthplace of Strabon) in the name of Ilduz Hatun, the wife of Öljaitü Khan (Muhammad Khodabandeh), who was on the throne between 1304-1316 (25,34).

**Figure 4. A,B:**
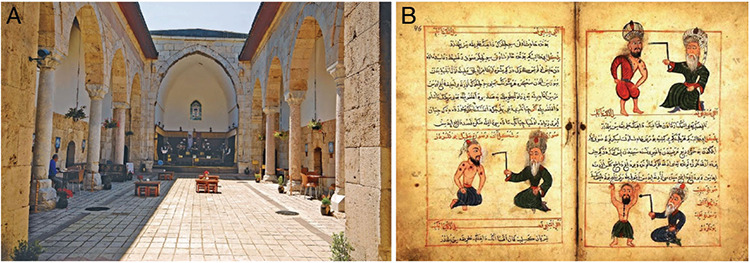
Intermediate period of İlhanlı sovereignty: Amasya, 1308. Inner courtyard with portico of the Amasya Darüşşifası (hospital), which was renewed on behalf of the wife of Olcaytu Han, İlduz Hatun. After the 2011 restoration, “Sabuncuoğlu History of Medicine and Surgery Museum” (a) (35); The manuscript written by Sabuncuoğlu Şerefeddin, a doctor from Amasya, about surgery; “Kitab-ül Cerrahiyye-I İlhaniye” (b) (36).

**A) 1308, Amasya Hospital:** In the inscription of the Amasya Darüşşifası, which is the most elegant hospital that has survived to the present day, it is written that it was built in the name of Ilduz Hatun by his free slave Anber bin Abdullah in H. 708 (1308) (Figure 4A).

However, it is mentioned in the Ottoman archives as “Sultan Alaeddin Darüşşifası” (there are documents showing that it was active until 1837). It is a typical Anatolian Seljuk work in terms of its architecture. Newly found documents also indicate Sultan I. Alâeddin Keykubat as its builder (1220-1237). Since the original foundation had not recovered, it was repaired during the period of the İlkhanians, who captured Amasya in 1308, and a new inscription was put in place. "Kitab-ül Cerrahiyye-i İlhaniyye” (1465), written by Sabuncuoğlu Şerefeddin (1385-1468), who was a chief physician for 14 years, was considered as the reference work of Ottoman physicians (1465) (Figure 4B). It was restored by the Municipality of Amasya in 2011 and opened as a “Sabuncuoğlu Tıp ve Cerrahi Tarihi Müzesi” (Sabuncuoğlu History of Medicine and Surgery Museum).

## LATE PERIOD (OTTOMAN PERIOD)

The Ottoman state was established in Western Anatolia (Bithynia), a former Byzantine land, as a small House affiliated to Konya Seljuks and Ilkhanians, and declared its independence in 1299. Expanded against Byzantium [Bursa (former Prusia) 1326 first capital], conquered Edirne (Adrianopolis) in 1361 and made it the second capital by passing through the Dardanelles to the European territory. After the conquest of Istanbul (Constantinopolis) (1453), it spread to three continents (Europe, Asia and Africa) under the order of the Empire (37). Physician training institutions emerged relatively late in the Ottoman state because experienced physicians from the Muslim principalities in Anatolia, from Iran, Azerbaijan, and the Islamic Middle East, flocked to this state in the ascension period. The Ottomans preserved the hospitals in Central and Eastern Anatolia and established 8 new hospitals based on Islamic principles in Western Anatolia and Thrace (5 of them are in Istanbul). This distribution, which is an expression of extreme centralization, shows that an absolute monarchy prioritizes the need for physicians for the palace and army. Only the Sultan and his family (mother or wifes; sultanas) were able to establish these “Medrese-i etıbba” type (educating more physicians) large size and large capacity rich foundations. Their foundations and inscriptions are preserved and their buildings are still standing - they are being used as health museums, libraries, or for educational purposes (two of those in Istanbul were destroyed by an earthquake and no attempts of renewal were made).

**Figure 5. A-C:**
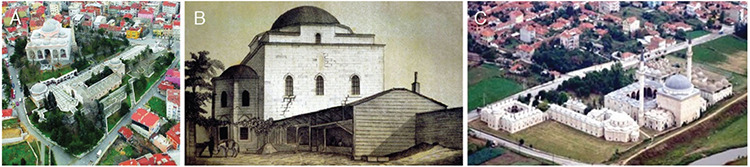
XIV^th^ century Ottoman Hospitals: Bursa, 1400. First medical school of Ottoman Empire: Bayezid’s I “Dar-üt-tıbb.” After the 2001 restoration, it continues to serve as “Bursa Darüşşifa Göz Merkezi” (Eye Hospital) (a) (39); İstanbul-Fatih, 1470. Darüşşifa Mescidi, which remains from Ottoman Empire’s first comprehensive education and health institution in İstanbul, Fatih Külliyesi (Islamic complex). (Engraving of A.G. Paspatis dated 1877, before it collapsed) (b) (40); Edirne, 1488. Darüşşifa and Medical Madrasah from Sultan Bayezid II Külliyesi (Islamic complex). Today, “Medical Museum”-Council of Europe Museum Award (2004), in UNESCO World Heritage temporary list (2016) (c) (41).

**A) 1400, Bursa Yıldırım Bayezid Medical School:** The first Medical School of the Ottoman Empire (under the name “Dâr-üt-tıbb”) was founded in Bursa, on May 12, 1400. It was located in a large islamic complex (Yıldırım Külliyesi) built by the fourth Ottoman monarch Bayezid I (Yıldırım). Patients were treated here for many years with its open courtyard and portico, 70 wards, and a staff of 25, as well as training physicians. Famous physicians who were affiliated with Islam served here. Since 2001, it has been allocated to a private foundation and continues to treat patients as the “Bursa Darüşşifa Göz Merkezi” (Eye Hospital) (Figure 5A).

**B) 1470, İstanbul Fatih Hospital:** Sultan Mehmet II, who conquered Istanbul (Constantinople) in 1453 and was briefly known as “Fatih,” is considered as the founder of the Ottoman Empire. Mehmet II, who made the city the capital, built a large islamic complex (mosque, mausoleum, 16 madrasahs, hospital, imaret, inn, library, Turkish bathhouse) between the years of 1463-1470 besides palaces, bazaars, harbors and military facilities. Fatih Darüşşifası (Fatih Hospital), which had 70 cells, 80 domes, and 200 staff, had its own masjid and bath. It was considered as the state's top-ranked “royal /imperial” hospital and medical school (today’s İstanbul Faculty of Medicine basis its foundation on the Fatih Darüşşifası (hospital) of 1470). However, despite the dangerous cracks the building suffered from during the Istanbul earthquakes (1509, 1557, 1754, 1766), it continued to provide healthcare services until 1824. The hospital part was demolished with the consent of the trustee, while the private masjid (“Masjid Masjid”) lasted until 1877 (Figure 5B). It was not rebuilt due to the presence of other hospitals in İstanbul and European-type Medical schools started being preferred. The hospital ruins apparently could be seen until 1970. Today, “Eski Şifahane Sokak” (street), which lies outside the northeast corner of the complex, is the heirloom of this magnificent work.

**C) 1488, Edirne Bayezid II Hospital:** Although Sultan Bayezid II (son of Fatih Sultan Mehmet) came to the throne in İstanbul, he did not forget Edirne, where he was born and grew up, and built an enormous complex on the coast of Tundzha River between 1484-1488 (mosque, inn, hospital and medical school, imaret, Turkish bathhouse, bridge and mill). Rich foundations donated for daily spending and annual repairs. The architectural plan of the hospital was unique and functional, and the inpatient section included a hexagonal plan, a dome with a lantern, a central space, patient rooms that could open up all around them, and ceilings. The water sound of the fountain in the middle, the chapters performed by the musical commitee on the bottom podium, and the fragrant flowers in the outer courtyard helped the patients to find spiritual peace (Evliya Çelebi). It was unprecedented in Turkish hospital architecture due to its inside-porched outside courtyard for outpatients with rooms for physicians and pharmacists opening up to the inner courtyard. Medrese-i etıbba was sharply separated (a square courtyard, surrounded by a gallery with columns, domed cells with furnaces to each of the 18 boarding students, and a large classroom with a library), yet it was located right next to the application hospital (38). Three foundations have been found and all three inscriptions can be read in the mosque. The hospital, which served until 1911, was closed in the Balkan War and World Wars, and it was allocated to the newly established Trakya University in 1984. The Hospital section was opened in 1997 and the Madrasah section was repaired and opened to visitors as a museum in 2008. It won the “Council of Europe Museum Award” in 2004 and entered the UNESCO World Heritage Temporary List in 2016. Situated in Turkey's European territory, it is the best preserved and most well-organized health museum (Figure 5C).

**Figure 6. A-C:**
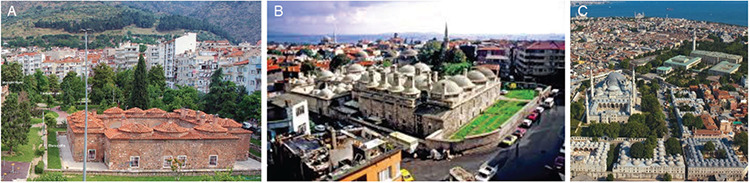
Hafsa Sultan, the mother of Magnificent Süleyman. Since 2013 “Medical History Museum” (a) (43); İstanbul-Haseki, 1551. Darüşşifa in Haseki Hürrem Sultan Külliyesi (Islamic complex) (on the left side), who is Magnificent Süleyman’s ex-wife (b) (44); İstanbul-Süleymaniye, 1557. The greatest Darüşşifa and Dar-üt-tıbb (medical faculty with the highest degree) of the Ottoman Empire, in the Külliye (Islamic complex) of Magnificent Süleyman (c) (45).

**D) 1539, Manisa Hafsa Sultan Hospital:** Ayşe Hafsa Sultan, the wife of Sultan Selim I and the mother of Suleiman the Magnificent, lived in Manisa for 8.5 years during the governorship of her son, got sick there, and was treated by the famous doctor Merkez Efendi. She is the first woman to take the title of “sultan” in Ottoman history. Although she moved to Istanbul Palace when her son took the throne in 1520, she started the construction of a large Islamic complex (“Sultaniye Kulliyesi”: mosque, madrasah, school, imaret) in Manisa in 1523. When she died in 1534 before completing, Suleiman the magnificent added a bathhouse (1538) and a hospital (1539) to the complex on behalf of his mother. This structure with three iwans without a porch and nine rooms around a rectangular open courtyard was located in the yard of the Sultaniye Mosque. According to the documents, 25 staff members received salaries from the foundation. According to Evliya Çelebi, “disciple physicians” were taught two days a week (1671). When Modern Hospitals were being established in the second half of the XIXth century, it assumed the role of “Bimarhane” (“madhouse” among the people) for the mentally ill. It was transferred to Celâl Bayar University in 1996 and opened to visitors as “Tıp Tarihi Müzesi” (History of Medicine Museum) in 2013 (Figure 6A).

In the XVI^th^ century, the Ottoman Empire experienced its strongest years in military and economic terms. For Suleiman, the Magnificent period (1520-1566) was his brightest period. In the said years, two Islamic complexes in the heart of the capital Istanbul had risen, as the works of the famous architect Sinan: one dedicated to Suleiman the Magnificent and the other on behalf of his beloved wife Hürrem Sultan.

**E) 1551, Haseki Hürrem Sultan Hospital:** First of all, the small-sized “Haseki Külliyesi” was built in the name of Hürrem Sultan (Europeans know it as “Roxelana”), who was the Sultan's wife through civil marriage and went everythere he went; this was the first work of Mimar Sinan. It was placed on the seventh hill (former Arkadius Forum) of the city, overlooking the Marmara Sea in the Old Women's Market, where female slaves were sold (Hürrem Sultan wanted to serve the women in need). After the first Mosque (1538), one year and 12 years later respectively Madrasah and a Primary School (1539) and imaret and a hospital (1551) were built. Hürrem Sultan died in 1558 and her tomb is in the Süleymaniye Mosque in a fenced-off burial area. Although the hospital is small in size, it has an original architectural plan that has not been seen before: an octagonal open courtyard, 6-quarters in two symmetrical sections, a double-domed iwan. The foundation of 1551 is in the Süleymaniye Library. It provided healthcare services until 1881 and was used as the Women's Hospital, “Nisa Tevkifhanesi,” Dormitory, and was renovated in 2011 (Figure 6B). Since 1976, it has been serving as the Training Center for the directorate of Religious Affairs and visitors are allowed in to it.

**F) 1557, Süleymaniye Hospital:** This enormous complex of Suleiman the Magnificent was built by Mimar Sinan in the historical center of the city between 1550 and 1557 [“Suleymaniye Kulliye”: Great Mosque, Shrines, Darulkurrâ (madrasah for reading Qur'an), Darülhadis (madrasah for reading Prophet Muhammed’s sayings, Primary School, four Madrasahs (Evvel, Sânî, Salis, Râbî), inn, Imaret, hospital, Medical Madrasah]. This Darüşşifa (hospital) was the largest imperial health institution seen in the Ottomans with two-courtyards (for patients and physicians), partly two storey, own bathhouse, bakery and medicine production (drug house), separate place for medical education (Dâr-üt-Tıbb, Medrese-i etıbba), and was superior to other hospitals (Figure 6C). It continued to function as a hospital until 1873, then it was used as a printing house, and today it is home to the Süleymaniye Manuscript Library, although it is closed to visitors. The adjacent Medicine Madrasah became the highest-ranking physician training institution of the empire and was combined with the “Süleymaniye Doğumevi” (Suleymaniye Maternity Hospital) built in 1946. The existing building of the Gynecology and Obstetrics Hospital was added to the Süleymaniye Manuscript Library when its health care services were moved to Zeytinburnu in 2009 and to Halkalı in 2011 (42).

In the years after Suleiman the Great, the Empire hit a period of stagnation and the construction of new health and welfare institutions became sparse and lost their former glory. However, until the beginning of the XVII^th^ century, islamic complexes containing some new hospitals were seen.

**Figure 7. A,B:**
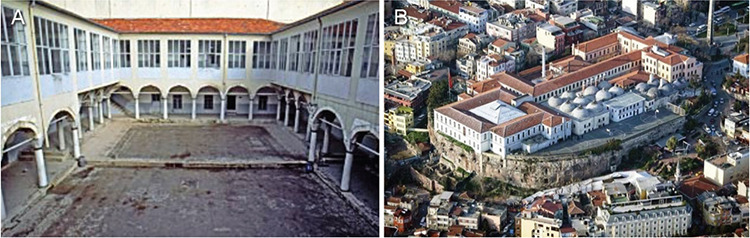
The last Hospitals of the Ottoman period: İstanbul-Üsküdar, 1583. Open courtyard of the Darüşşifa of the Külliye (Islamic complex) (2010), built on the Anatolian side in the Topbaşı district on behalf of Atik Valide Sultan (Nurbanu Sultan, the mother of Murad III). Today “Fatih Sultan Mehmet Foundation University Faculty of Literature” (a) (46); İstanbul-Sultanahmet, 1621. Bath ruins (2010) from last Ottoman Darüşşifa (hospital) in Sultan Ahmet I Külliyesi (Islamic complex). It was built on the Shendone extension of the Hippodrome of Constantinople (b) (47).

**G) 1583, İstanbul-Üsküdar Atik Valide Sultan Hospital:** The Bride of Suleiman the Magnificent, Sultan Selim's wife, and Nurbanu Sultan who was the bride of Suleiman the Magnificent, Sultan Selim's wife and mother of Sultan Murad III, donated great charity to the Anatolian side of İstanbul (Toptaşı district of Üsküdar) in 1583 (Atik Valide Sultan Külliyesi: Mosque, Madrasa, Darülkurrâ, Darülhadis, Imaret, Hospital, Library, Double Turkish Bathhouse and Halveti Tekke). In this last known work of Mimar Sinan, the hospital section had a rectangular courtyard and two floors and has undergone many changes in the past years. In 1805, during the reign of Sultan Selim III, it was transferred to the barracks of Nizam-ı Cedid soldiers, then to the Military Hospital, to the biggest mental hospital of Istanbul under the name of “Toptaşı Bimarhanesi” between 1865-1927, to Imam-Hatip High School in 1970, and to the Fatih Sultan Mehmet Foundation University in 2010. In the restoration of 2011-2013, its originality was altered by concrete and glass panes and the Faculty of Letters of the relevant university was established there (Figure 7A).

**H) 1621, Sultan Ahmet I Hospital:** Sultan Ahmet I, who ascended to the throne at the age of 14 in 1603, had a very ambitious project when he was 20 years old and started the construction of a large islamic complex (“Sultanahmet Külliyesi”: mosque, madrasah, darülkurrâ, shrine, bazaar, baths, inn, imaret and hospital) in the center of the historical “Constantinopolis” across Hagia Sophia (1609). Its architect was Sedefkâr Mehmet Ağa. The best piece of this complex was the Sultanahmet Mosque with 6 minarets and 16 balconies (Mavi Cami, Blue Mosque). when it had been completed in 1617, it was the most magnificent mosque in Istanbul, but at the end of the same year, Sultan Ahmet I died at the age of 28 due to a febrile diseaese. Other complex units mentioned in his foundation of 1614 was completed by his son Sultan II Osman (Genç Osman) (Figure 7B).

The buildings in this complex were in a non-contiguous scattered order because they were built on the remains of the Great Palace of the Roman-Byzantine Constantinopolis and partly on the Hippodrome (Hippodrome) tribunes. For this reason, “Imaret” and “Darüşşifa” (hospital) units were located on the Sfendon (Sphendone) ledge, which is the continuation of Meydani in the direction of Marmara. When the Hippodrome area for horse car races came up short, Roman architects found the solution of “sphendone” (round extension, ergo “the back of the goal net” of football stadiums), building it on the dip slope going down using Roman bricks and mortar, enormous carrier arches, galleries, and corners. This 1700-year-old fortification still carries multi-storey buildings on top of it, despite the earthquakes. After the conquest of İstanbul (in 1454), “Kılıçhane Binası” (building for forging swords) was built in this area and the foundry and iron processing workshops of the army were located here. In 1615, Kılıçhane was moved to another place and the Imaret of the Sultanahmet Külliyesi (kitchen, pantry, bakery, dining hall) and the Hospital (square plan, porched courtyard, single row domed spaces, own masjid and bath) were built (1621). This last hospital of the Ottoman State was allocated to mental patients in the beginning of the XIXth century (Sultanahmet Bimarhanesi), then became a military sewing room in 1846, “Islah-ı Sanayi Mektebi” (industrial school)in 1868, and is today “Sultanahmet Meslekî ve Teknik Anadolu Lisesi” (Sultanahmet Vocational and Technical Anatolian High School). After the additional construction and repairs, only the bath, entrance door, and marble bowls of the pool in the courtyard remained from the hospital. Contruction of the imaret on the front that was facing the hippodrome square was abandoned in 1883 with the construction of “Hamidiye Ticaret Mekteb-i Âlisi” (Neo-Ottoman and Art Nouveau style, architect Raimondo D’Aronco), and it was transferred to Istanbul Academy of Economic and Commercial Sciences in 1959; in 1982, it became the Sultanahmet Campus (Rectorate and Senate Hall) of Marmara University.

**Figure 8:**
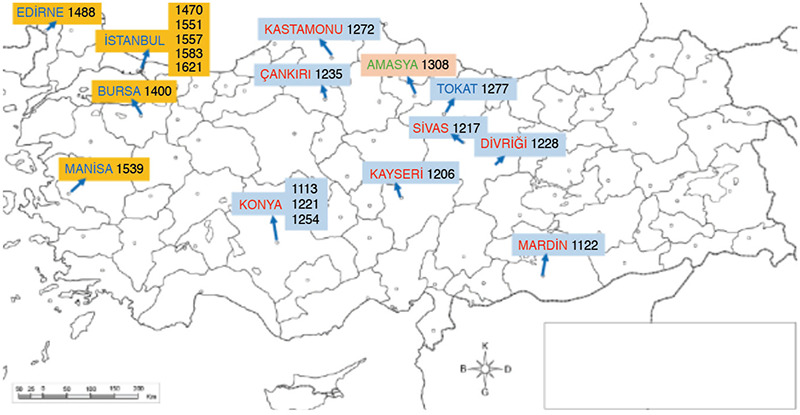
The distribution of islamic health institutions that trained physicians in the geography of today’s Turkey (48).

According to the order that was established, 12 of the 19 health facilities (Figure 8) which we provided brief information have been restored by various institutes (General Directorate of Foundations, Municipalities, Universities) and used as follows: 4 of which are used as Museum of Health and Medical Education (Kayseri, Amasya, Edirne and Manisa), 4 as general Museum Buildings (Mardin, Sivas, Divrigi, Tokat), 1 as a modern health clinic (Bursa), 1 as a modern university faculty (İstanbul-Üsküdar), 1 as a Manuscript Library (İstanbul-Süleymaniye), and 1 as a Training Center (İstanbul-Haseki). Unfortunately, there are either no traces left or only trivial remnants is found of the 3 hospitals in the capital of the Seljuk Sultanate, 2 hospitals in the capital of the Ottoman Empire (Fatih, Sultanahmet), 1 hospital in Çankırı, and 1 hospital in Kastamonu.

In the Ottoman Empire, very detailed population and real estate counts were made, recorded periodically in books (cadastral record books), and maintained in a central archive. The edicts, charts, donations and foundations signed by the sultans have survived to the present day. “Kadi registers” (court minutes, notary contracts) belonging to every region of the empire were also collected in the central archive. There was no other empire that kept such rigorous and detailed records and preserved them for centuries. Today, Millions of documents are still be read and classified. Since it is also open to researchers from all over the world.

For this reason, new information about the health institutions whose buildings have been burned down and forgotten by local people can be obtained from the above-mentioned records. We can collect information about health institutions such as Darüşşifa or hospitals, which were once active in Anatolia (Erzurum, Erzincan, Niksar, Aksaray, Harput, Silvan, Kütahya) and in the Balkans (Thessaloniki, Skopje, Sofia, Sarajevo, Belgrade, Budin), thanks to the reading and publication of these documents by experts.
